# Negative differential resistance and carrier transport of electrically bistable devices based on poly(*N*-vinylcarbazole)-silver sulfide composites

**DOI:** 10.1186/1556-276X-9-128

**Published:** 2014-03-19

**Authors:** Jiantao Li, Aiwei Tang, Xu Li, Yapeng Cao, Miao Wang, Yu Ning, Longfeng Lv, Qipeng Lu, Yunzhang Lu, Yufeng Hu, Yanbing Hou, Feng Teng

**Affiliations:** 1 Key laboratory of Luminescence and Optical Information, Ministry of Education, School of Science, Beijing JiaoTong University, Beijing 100044, China; 2 Institute of Optoelectronic Technology, Beijing JiaoTong University, Beijing 100044, China; 3 Department of Chemistry, School of Science, Beijing JiaoTong University, Beijing 100044, China

**Keywords:** Bistable device, Ag_2_S nanocrystals, PVK, NDR effects, Charge trapping

## Abstract

An electrically bistable device has been fabricated based on poly(*N*-vinylcarbazole) (PVK)-silver sulfide (Ag_2_S) composite films using a simple spin-coating method. Current–voltage (*I-V*) characteristics of the as-fabricated devices exhibit a typical electrical bistability and negative differential resistance (NDR) effect. The NDR effect can be tuned by varying the positive charging voltage and the charging time. The maximum current ratio between the high-conducting state (ON state) and low-conducting state (OFF state) can reach up to 10^4^. The carrier transport mechanisms in the OFF and ON states are described by using different models on the basis of the experimental result.

## Background

Organic electrically bistable devices have aroused extensive interests due to their unique advantages such as simple-fabrication process, large memory density, and lower power consumption [1-3]. A wide variety of materials, including conjugated polymers, small organic molecules and inorganic nanocrystals, have been applied to obtain better device performance [4-6]. Among different candidates for electrically bistable devices, colloidal inorganic nanocrystals have been studied extensively due to their unique chemical and physical properties. To date, some different types of inorganic nanocrystals, such as ZnO, Cu_2_S, and CdSe/ZnS have been embedded into polymers to fabricate electrically bistable devices, which have exhibited clear electrical bistabilities [7-10]. These nanocrystals mentioned above, however, have their intrinsic defects, such as toxicity and instability, which limit their further applications [11,12]. In the electrically bistable devices based on inorganic nanocrystals, NDR effects standing for the current decreasing with the increasing bias voltage have often been observed, which have aroused much attention since it is considered to be a key feature for their conduction system [13-15]. As promising optoelectronic candidates, Ag_2_S nanocrystals have the advantages of less toxic and good stability, which are still rarely seen in the reports of organic electrically bistable devices.

In this letter, an electrically bistable device has been fabricated based on the composites containing spherical Ag_2_S nanocrystals and PVK using a simple spin-coating method. Current–voltage (*I-V*) measurements as well as retention and reproducibility tests have demonstrated that the devices show good electrical bistability and stability. The NDR effects have been studied by applying different positive charging voltages and the charging time, which can be attributed to the charge trapping/detrapping process in the Ag_2_S nanocrystals. Moreover, the carrier transport mechanism has been described based on the *I-V* results.

## Methods

**Figure 1:**
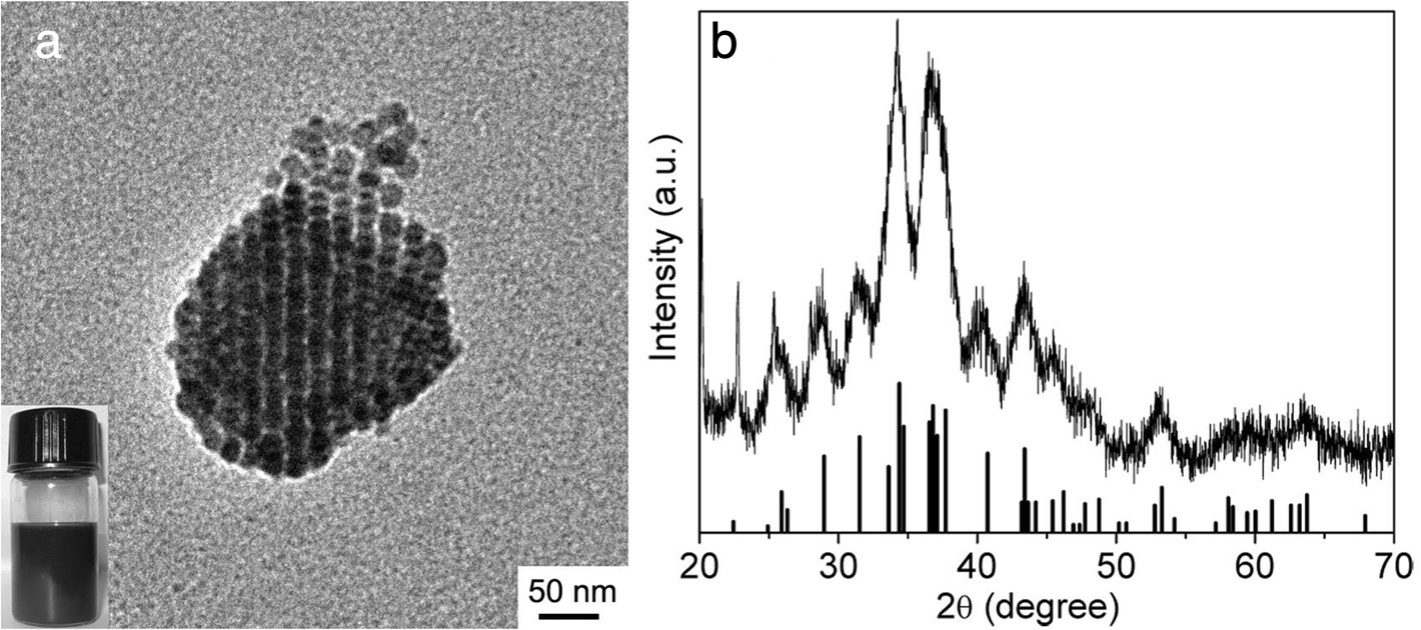
**TEM image and XRD patterns and standard diffraction lines of Ag**_**2**_**S.** TEM image of resultant Ag_2_S nanocrystals **(a)** and XRD patterns of Ag_2_S nanocrystals and standard diffraction lines of monoclinic Ag_2_S **(b)**.

The Ag_2_S colloidal nanocrystals used in this study were prepared according to our previous report [16]. The transmission electron microscopy (TEM) image of the as-obtained Ag_2_S nanocrystals shown in Figure 1a exhibits a spherical shape with an average diameter of 6.4 ± 0.7 nm. Figure 1b shows the X-ray diffraction (XRD) patterns of the Ag_2_S nanocrystals, and all the diffraction peaks can be indexed to the monoclinic Ag_2_S phase (JCPDS card: no. 14-0072).

The electrically bistable devices were fabricated on glass substrates pre-coated with an indium-tin-oxide (ITO) anode, which were alternately cleaned by deionized water, acetone, and ethanol in an ultrasonic environment. Afterwards, the poly(3,4-ethylenedioxythiophene)/poly-(styrene-sulfonate) (PEDOT/PSS) was spin-coated onto the substrate and was annealed at 150°C for 20 min, which could smooth the ITO surface and improved the device stability by hindering oxygen and indium diffusion through the anode. The PVK and Ag_2_S nanocrystals were mixed and dissolved in chlorobenzene solution with a mass ratio of 1:1. The solution would further form the active layer by the spin-coating method. Finally, a top Al electrode layer of 200 nm thickness was deposited onto the top surface by thermal evaporation under the vacuum of about 1 × 10^−6^ torr.

## Results and discussion

**Figure 2:**
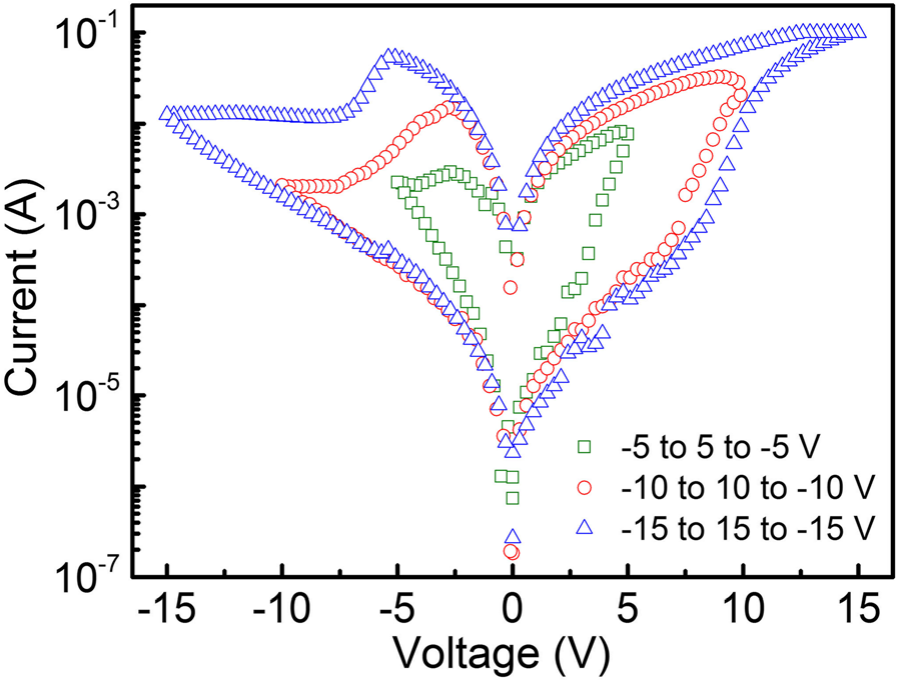
***I-V *** **characteristics of device with a structure of ITO/PEDOT:PSS/Ag**_**2**_**S PVK/Al under different sweeping voltage ranges.**

The *I-V* characteristics of the devices with a structure of ITO/PEDOT:PSS/Ag_2_S:PVK/Al under different sweeping voltages are shown in Figure 2. The voltage scan sweeps −5 to 5, −10 to 10, and −15 to 15 V, respectively. All the *I-V* curves of the devices under different sweeping voltages exhibit a typical electrical bistability. The magnitude of the *I-V* hysteresis increases with increasing maximum sweeping voltages, and the ON/OFF current ratio of the device can approach 10^4^. Herein, we take the *I-V* result under the sweeping voltage from −15 to 15 V as an example to describe the electrical hysteresis process. When the sweeping voltage exceeds a certain threshold, namely *V*_on_ (about 8 V), the current increases rapidly, which indicates that the conducting state transforms from OFF to ON state. When the sweeping voltage scans from 0 to −15 V, the current reaches its maximum at a certain voltage (about −6 V), which is labeled as *V*_off_ (the voltage region where the NDR effect takes place), and then decreases quickly with the increasing reverse voltage, which is a typical NDR behavior. As a result, the conducting state changes from ON to OFF state. Considering that there is no obvious hysteresis observed in the device using only PVK as active layer we suggest that the Ag_2_S nanocrystals play a significant role in the electrical bistability. Furthermore, it can be seen in Figure 2 that the absolute value of *V*_off_ increases with the increasing magnitude of the sweeping voltage, indicating that there might be a certain connection between the NDR effect and the sweeping voltages.

**Figure 3:**
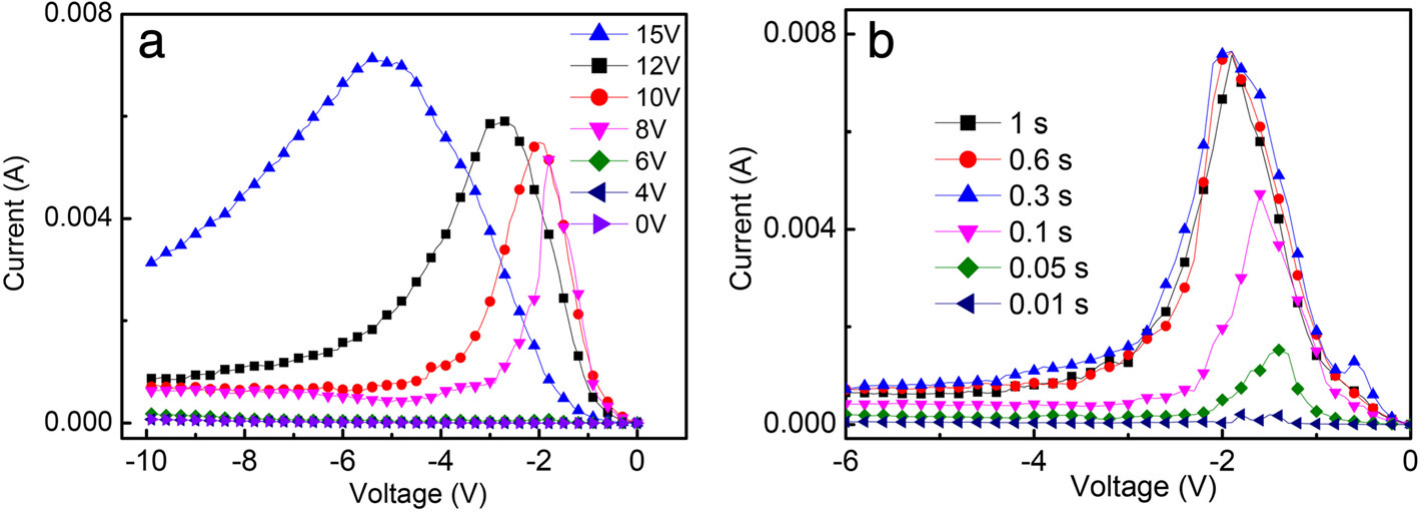
**NDR behaviors of device with ITO/PEDOT:PSS/Ag**_**2**_**S:PVK/Al measured under different (a) positive charging voltages and (b) charging time.**

To get a better understanding of the NDR effects in the bistable devices, the *I-V* characteristics of the device under different positive charging voltages (0 to 15 V) were measured. In this process, the device was firstly charged by a certain voltage for 0.1 s, and then the *I-V* curves were measured in the negative sweeping region. Figure 3a depicts the *I-V* curves under different positive charging voltages, and it can be seen that the NDR behavior is not observed until the positive charging voltage reaches up to 8 V, which just equals to the value of *V*_on_. This phenomenon can be well explained by a charge-trapping mechanism [17-19]. In this hypothesis, the electrons will overcome the energy barrier and occupy the traps in the organic matrix under a positive voltage, resulting in the change of the conducting states of the device. In contrast, the limited charges can be expelled out of the trap centers under a proper reverse voltage, resulting in the recovery of the conducting state and the appearance of the NDR behavior. Correspondingly, the NDR effect will not appear if the positive charging voltage is not large enough, which is just what happened in our test. Furthermore, as shown in Figure 3a, the absolute value of *V*_off_ increases with the increasing charging voltage. As an example, the *V*_off_ jumps from −2 to −5 V when the charging voltage increases from 10 to 15 V. This relationship between the absolute value of *V*_off_ and the charging voltage reveals the fact that higher reverse voltages favor the charges release captured in deeper traps under higher charging voltages. Therefore, the NDR effects represent a discharge process, while the positive voltages play an important role of the charging.

Moreover, the NDR effects under different charging time (0.01 to 1 s, 10 V) were also studied, and the corresponding *I-V* characteristics in the NDR region are given in Figure 3b. It can be seen that the absolute current value at *V*_off_ increases as the charging time is increased from 0.01 to 0.3 s. This indicates that more charges have been seized by trap centers with longer charging time, which results in larger discharging current in the NDR region. However, the *I-V* characteristic saturates when the charging time of the applied voltage reaches 0.3 s, indicating the traps in device will be completely occupied after a certain charging time, which may be attributed to an oxidation process related to the oxygen vacancies on the surface of Ag_2_S nanoparticles [20].

**Figure 4:**
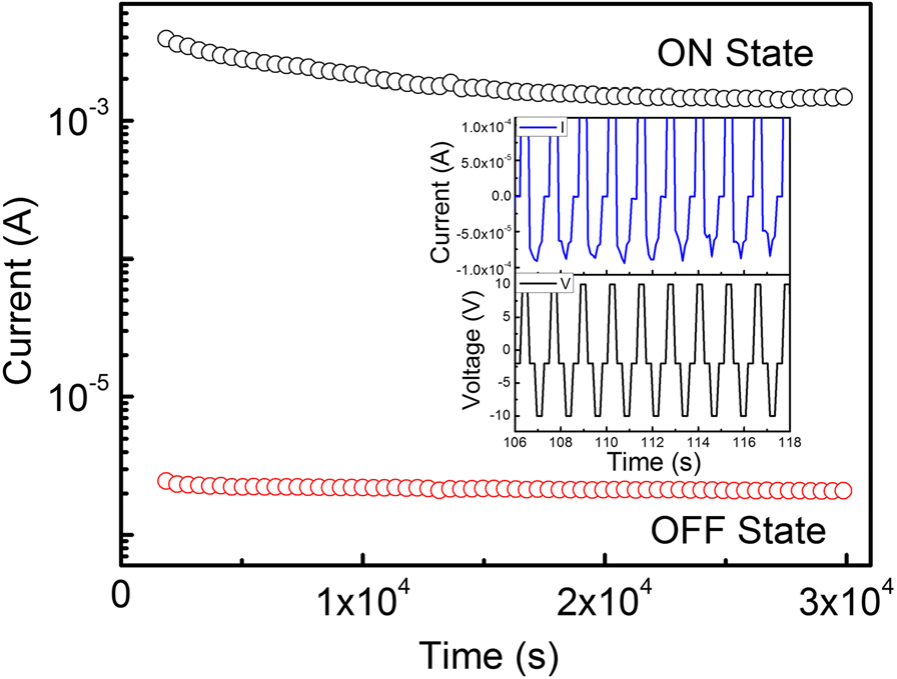
**Retention ability of electrically bistable devices under the sweeping voltage of 1 V.** The inset shows switching performance of device during a programmed ‘write-read-erase-read’ sweeping sequence.

Apart from the ON/OFF current ratio, the retention ability and switching endurance are two other important parameters for a typical electrically bistable device. The current in the ON and OFF states of the electrically bistable devices can be sustained under a constant voltage of 1 V for up to 8 h, which is given in Figure 4. It can be seen that the ON/OFF ratio undergoes a slight decline in the beginning and remains at about 10^3^ during the rest time of the test, indicative of a reliable memory retention performance. The little degradation of the ON/OFF ratio is mainly from the decrease of the ON state current, which is probably associated with the unstable interfacial contact between the surfaces of the organic matrix and Ag_2_S nanocrystals [5]. To test the reproducibility of the devices, a programmed voltage sequence of 10, −2, −10, −2 V was applied to the device circularly to simulate the write-read-erase-read process, and the result is depicted in the inset of Figure 4. The ON/OFF current ratio is more than two orders of magnitude and the current changes disciplinarily and reproducibly during the write-read-erase-read switching sequence.

**Figure 5:**
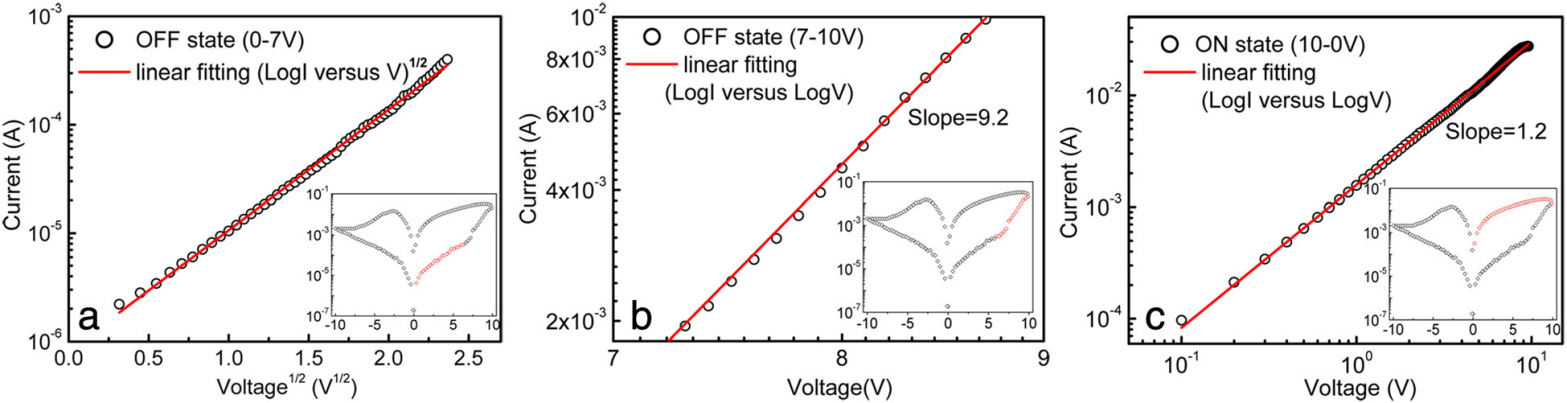
**Experimental results (open cycle) and theoretical linear fitting (solid line) of *I-V *characteristics in positive voltage region. (a)** Linear relationship of log*I* versus log*V*^1/2^ in the voltage region of 0 to 7 V (OFF state); **(b**) linear fit in double logarithmic scale in the voltage region of 7 to 10 V (OFF state); **(c)** linear fit in double logarithmic scale at voltage region of 10 to 0 V (ON state).

To clearly understand the carrier transport mechanism in the electrically bistable devices, we have fitted the experimental *I-V* curves in ON and OFF states by using some theoretical models of organic electronics. Figure 5a,b shows the experimental results and the linear fitting for the OFF state in the positive voltage region. As shown in Figure 5a, the experimental *I-V* curve in the voltage region of 0 to 7 V can be well fitted by the thermionic emission model (log*I∝V*^*1/2*^), indicating that the current is dominated by the charge injection from the electrodes [21]. However, when the applied voltage sweeps from 7 to 10 V, the log*I*-log*V* characteristics shown in Figure 5b exhibit a large linear slope of 9.2, which is consistent with a trap-controlled space charge limit (TCLC) model (*I∝V*^*α*^, *α > 2*) [22]*.* The fitting result indicates that when the applied voltage surpasses *V*_on_, the charges will break the energy barrier and can be captured in the traps by the Ag_2_S nanospheres with an exponential distribution in the forbidden gap.

In contrast, the experimental *I-V* result in ON state can be well described by an ohmic model, which is depicted in Figure 5c. It can be seen that a distinct linear relationship between log*I* and log*V*, with a slope of 1.2 in the positive (10 to 0 V) region. The theoretical fitting illustrates that the current of the device is approximately proportional to the applied voltages, which is close to the Ohmic law (*I∝V*) [23]. The results reveal that the traps in the Ag_2_S nanospheres have filled by the carriers during the aforementioned TCLC process. The trapped carriers lead to the rise of the internal electrical field at the Ag_2_S/PVK interface, which can change the conductivity of the device. All the results of the theoretical fitting are consistent with the charge trapping mechanism.

## Conclusions

In summary, organic bistable devices based on Ag_2_S-PVK composites were fabricated by a simple spin-coating method. Obvious electrical bistability and NDR effects have been observed in the devices due to the existence of the Ag_2_S nanospheres. The NDR effects can be controlled by varying the charging voltages and charging time. The maximum ON/OFF current ratio can reach up to 10^4^. The carrier transport can be described in terms of the organic electronic models, and the carrier transport mechanism alters from the thermionic emission to the ohmic model during the transition from OFF state to ON state, which is closely associated with the charge trapping/detrapping process in the Ag_2_S-PVK composites.

## Competing interests

The authors declare that they have no competing interests.

## Authors' contributions

JL designed the study, prepared the device, carried out the electrical measurement. JL and AT wrote the manuscripts. AT, FT, and Y Hou conceived and designed the study. MW performed the TEM and XRD test. XL and YC participated in the fabrication of the device. LL, YN, QL, Y Hu, and YL participated in interpreting the results. All authors read and approved the final manuscript.

## References

[B1] Yang Y Ouyang J Ma L Tseng RJH Chu CW Electrical switching and bistability in organic/polymeric thin films and memory devices Adv Funct Mater 2006 9 1001 10.1002/adfm.200500429

[B2] Mukherjee B Mukherjee M Nonvolatile memory device based on Ag nanoparticle: characteristics improvement Appl Phys Lett 2009 9 173510 10.1063/1.3127233

[B3] Shim JH Jung JH Lee MH Kim TW Son DI Han AN Kim SW Memory mechanisms of nonvolatile organic bistable devices based on colloidal CuInS_2_/ZnS core–shell quantum dot – poly(*N*-vinylcarbazole) nanocomposites Org Electron 2011 9 1566 10.1016/j.orgel.2011.05.023

[B4] Ouyang JY Chu CW Szmanda CR Ma LP Yang Y Programmable polymer thin film and non-volatile memory device Nat Mater 2004 9 918 10.1038/nmat1269 15568028

[B5] Ma L Liu J Pyo S Yang Y Organic bistable light-emitting devices Appl Phys Lett 2002 9 362 10.1063/1.1436274

[B6] Liu JQ Yin ZY Cao XH Zhao F Lin AP Xie LH Fan QL Boey F Zhang H Huang W Bulk heterojunction polymer memory devices with reduced graphene oxide as electrodes ACS Nano 2010 9 3987 10.1021/nn100877s 20540553

[B7] Li FS Son D Ham JH Kim BJ Jung JH Kim TW Memory effect of nonvolatile bistable devices based on CdSe/ZnS nanoparticles sandwiched between C60 layers Appl Phys Lett 2007 9 162109 10.1063/1.2801357

[B8] Li FS Cho SH Son DI Park KH Kim TW Multilevel nonvolatile memory effects in hybrid devices containing CdSe/ZnS nanoparticle double arrays embedded in the C60 matrices Appl Phys Lett 2008 9 102110 10.1063/1.2898163

[B9] Son DI You CH Jung JH Kim TW Carrier transport mechanisms of organic bistable devices fabricated utilizing colloidal ZnO quantum dot-polymethylmethacrylate polymer nanocomposites Appl Phys Lett 2010 9 013304 10.1063/1.3454774

[B10] Tang AW Teng F Qian L Hou YB Wang YS Electrical bistability of copper (I) sulfide nanocrystals blending with a semiconducting polymer Appl Phys Lett 2009 9 143115 10.1063/1.3243981

[B11] Hardman R Environ A toxicologic review of quantum dots: toxicity depends on physicochemical and environmental factors Health Perspect 2006 9 165 10.1289/ehp.8284 PMC1367826 16451849

[B12] Selivanov EN Gulyaeva RI Vershinin AD Thermal expansion and phase transformations of copper sulfides Inorg Mater 2007 9 573 10.1134/S0020168507060027

[B13] Wang ML Sun XY Zheng XY Li N Gao XD Ding BF Ding XM Hou XY Loss and recovery of bistability of organic bistable devices Org Electron 2009 9 965 10.1016/j.orgel.2009.05.004

[B14] Xie M Aw KC Langlois M Gao W Negative differential resistance of a metal-insulator-metal device with gold nanoparticles embedded in polydimethylsiloxane Solid State Commun 2012 9 835 10.1016/j.ssc.2012.02.023

[B15] Bozano LD Kean BW Deline VR Salem JR Scott JC Mechanism for bistability in organic memory elements Appl Phys Lett 2004 9 607 10.1063/1.1643547

[B16] Wang M Wang Y Tang AW Li X Hou YB Teng F Optical properties and self-assembly of Ag_2_S nanoparticles synthesized by a one-pot method Mater Lett 2012 9 108

[B17] Ma L Pyo S Ouyang J Xu Q Yang Y Nonvolatile electrical bistability of organic/metal-nanocluster/organic system Appl Phys Lett 2003 9 1419 10.1063/1.1556555

[B18] Simmons JG Verderber RR New conduction and reversible memory phenomena in thin insulating films Proc R Soc Lond A 1967 9 77 10.1098/rspa.1967.0191

[B19] Cho B Song S Ji Y Kim TW Lee T Organic resistive memory devices: performance enhancement, integration, and advanced architectures Adv Funct Mater 2011 9 2806 10.1002/adfm.201100686

[B20] Verbakel F Meskers SCJ Janssen RAJ Electronic memory effects in diodes of zinc oxide nanoparticles in a matrix of polystyrene or poly (3-hexylthiophene) Appl Phys Lett 2006 9 102103 10.1063/1.2345612

[B21] Burroughes JH Jones CA Friend RH New semiconductor device physics in polymer diodes and transistors Nature 1988 9 137 10.1038/335137a0

[B22] Çakar M Güllü Ö Yildirim N Türüt A Electrical analysis of organic interlayer based metal/interlayer/semiconductor diode structures J Electron Mater 1995 9 38

[B23] Kapoor AK Jain SC Poortmans J Kumar V Mertens R Temperature dependence of carrier transport in conducting polymers: similarity to amorphous inorganic semiconductors J Appl Phys 2002 9 3835 10.1063/1.1506394

